# A threshold mechanism ensures minimum-path flow in lightning discharge

**DOI:** 10.1038/s41598-020-79463-z

**Published:** 2021-01-11

**Authors:** Franco Blanchini, Daniele Casagrande, Filippo Fabiani, Giulia Giordano, David Palma, Raffaele Pesenti

**Affiliations:** 1 Dipartimento di Matematica, Informatica e Fisica, Università di Udine, 33100 Udine, Italy; 2 Dipartimento Politecnico di Ingegneria e Architettura, Università di Udine, 33100 Udine, Italy; 3 Department of Engineering Science, University of Oxford, OX1 3PJ Oxford, United Kingdom; 4 Dipartimento di Ingegneria Industriale, Università di Trento, 38123 Povo, TN Italy; 5 Dipartimento di Management, Università Ca’ Foscari, 30121 Venezia, Italy

**Keywords:** Applied mathematics, Electrical and electronic engineering

## Abstract

A well-known property of linear resistive electrical networks is that the current distribution minimizes the total dissipated power. When the circuit includes resistors with nonlinear monotonic characteristic, the current distribution minimizes in general a different functional. We show that, if the nonlinear characteristic is a threshold-like function and the current generator is concentrated in a single point, as in the case of lightning or dielectric discharge, then the current flow is concentrated along a single path, which is a minimum path to the ground with respect to the threshold. We also propose a dynamic model that explains and qualitatively reproduces the lightning transient behavior: initial generation of several plasma branches and subsequent dismissal of all branches but the one reaching the ground first, which is the optimal one.

## Introduction

In lightning or gas electrical discharge, the current flow is essentially concentrated along a single path. Under very slow motion it can be seen that lightning starts by generating several branches and then develops by dismissing all of them but a single one, along which the energy is discharged^1^. This phenomenon has been deeply investigated. Several types of lightning are known, which are carefully described, e.g., in^2,3^. As far as the numerical modeling of the phenomenon is concerned, computational models for lightning simulation have been proposed in^4–7^, while the fractal nature of lightning discharge has been investigated in^8–11^. Detailed surveys on the subject are also available; see, e.g.^12,13^.

Here, we do not investigate the whole phenomenon in its complexity. We rather focus on a specific question about path formation in lightning discharge: we are interested in the initial phase of the process, when the lightning path is formed. Also, we consider the ideal case in which the lightning source is a single point and the final destination is a zero-potential ground. This type of lightning, classified as Category 1 Lightning, “is the most common cloud-to-ground lightning. It accounts for over 90% of the worldwide cloud-to-ground flashes”^3^. Cloud-to-ground lightning begins with an initial breakdown and the consequent creation of a ionized channel, the *stepped leader*, which generates several branches. Once the stepped leader is close to the ground, it may be approached by channels originating from the ground, the *connecting leaders*. When the stepped leader finally connects the ground to the cloud, the *return stroke* is triggered, which is a ground-potential upward wave^2,3,14^. After the return stroke reaches back the cloud, the main branch reaching the ground is crossed by a long-duration discharging current: the *continuing currents*. In the meanwhile, the secondary branches originally established by the stepped leader are depleted: the continuing currents flow along the main path only^2^.

Why is the lightning current eventually concentrated along a single path? Does this path enjoy any optimality property?

To mathematically address these questions, we consider an idealized model based on the assumption that lightning is mainly due to a dielectric breakdown of the air (gas in the case of discharges). The current–voltage diagram of a gas is characterized by two regions: for all the voltage values belonging to a symmetric interval around the origin, the current is very low (high resistivity); for voltage values outside this interval, the current becomes very large (low resistivity). The voltage value corresponding to the ends of the interval is called *breakdown threshold*. Then, we consider an ideal characteristic with conductivity approaching infinity when the electric field is larger than a threshold^5,15^.

Lightning path can be interpreted as the solution of an optimization problem over a network. To formulate the problem, we consider a graph describing an electrical network, where capacitances and resistors with possibly nonlinear characteristic are associated with the links. The grid model we use is akin to that proposed in^5^, Eq. 5, which is the discretized version of nonlinear field equations^5,15^. We show that the steady-state solution minimizes a convex functional that, in the special case of linear resistors, turns out to be the total dissipated power. Conversely, if the resistor characteristic is a threshold-like function, the steady-state solution becomes the *minimum path*, where each current link is weighted by its local threshold voltage; hence, the optimal path is the “minimum-threshold path”. Our main result is supported by a theorem and reinforced by simulations of randomly generated graphs with random threshold values, showing that the transient behavior of the model can faithfully reproduce the qualitative lightning evolution (see “Methods”).

**Figure 1:**
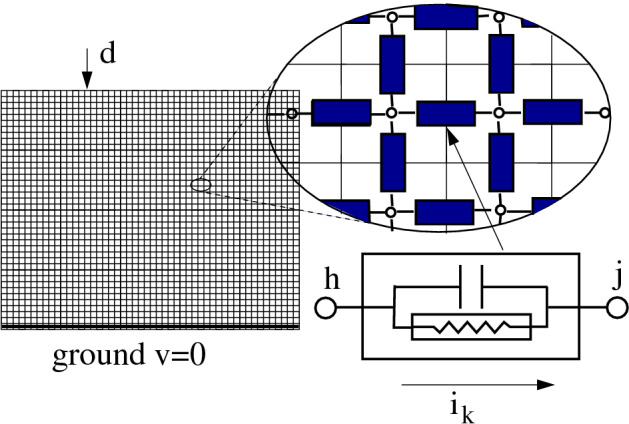
Electrical grid model. The electrical grid model with capacitances and possibly nonlinear resistors connecting adjacent terminals. In the graph representation, each terminal is associated with a node and each electric component with a link.

## Results

How does lightning optimize its path? To build a model, we consider the electrical grid network in Fig. 1, with a capacitive and a possibly nonlinear resistive effect between adjacent terminals. Ground terminals are connected among them with zero resistance (ideal conductor ground) and the ground potential is $v=0$. The network is associated with a graph where the $n+1$ nodes represent the terminals and the links represent the electric impedances. In particular, node *n* corresponds to the zero-potential ground, while at the source node 0 a current generator is applied, with its other terminal grounded, inducing an input current *d* that enters the network.

As shown in Fig. 1, each link is assumed to be the parallel connection of a capacitor and a possibly nonlinear resistor. Injecting a current *d* in a node of the network leads, after a transient, to a steady state in which the currents flow only through the resistors. If these are linear, the steady-state solution corresponds to the current distribution that minimizes the total dissipated power^16^:

1 $$\begin{aligned} P_{tot}=\sum _{k} R_k \bar{\imath }_k^2 \, \rightarrow \min , \end{aligned}$$

where $R_k$ is the resistance value associated with link *k* and $\bar{\imath }_k$ is the steady-state current flowing through it.

**Figure 2:**
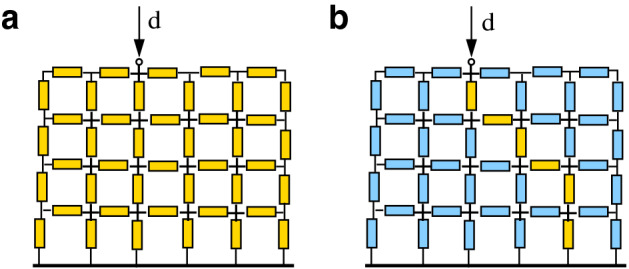
Electrical current distribution at steady state. The current is scattered in the case of linear resistances (**a**) and concentrated along a single path in the case of threshold characteristics (**b**), when the current flows along the path that minimizes the sum of the dielectric rigidities of its links. Yellow (resp. blue) means presence (resp. absence) of flowing current.

In this minimum power configuration, steady-state currents are scattered all over the network (as in Fig. 2a). In phenomena such as lightning, the situation is completely different^2,3^: after a transient, lightning “chooses” a single path (as in Fig. 2b). Why and how is this single path chosen?

In our model, denoting by $v_h$ and $v_j$ the potentials at the extreme nodes of link *k*, the resistance obeys the nonlinear law

$$\begin{aligned} R_k^{th}={\left\{ \begin{array}{ll} (v_h-v_j)/ i_k = \infty , &{}\quad \text{ if }\, |v_h-v_j| < V_k,\\ (v_h-v_j)/ i_k= 0, &{}\quad \text{ if }\, |v_h-v_j|\ge V_k, \end{array}\right. } \end{aligned}$$

where $V_k$ is the value of the threshold. We name such a threshold value *local* dielectric rigidity of link *k*. This value is defined as the minimum potential difference between two adjacent nodes (namely, two nodes connected by a link) necessary to induce a current flow.

**Figure 3:**
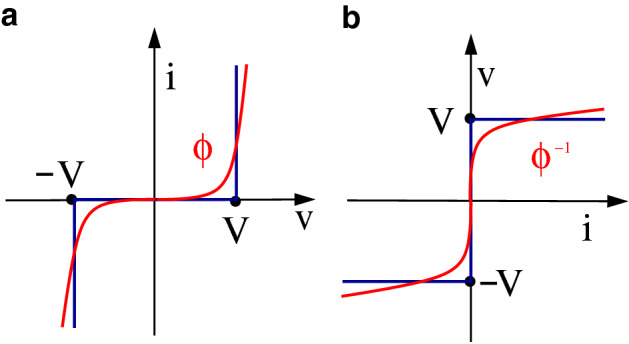
Nonlinear current–voltage and voltage–current characteristics. We denote by *v* voltage and by *i* current. Red: a generic threshold-like current–voltage characteristic $\phi$, with threshold *V* (**a**) and its inverse $\phi ^{-1}$ (**b**). Blue: the ideal, sharp threshold characteristics, which can be seen as the limit of a sequence of sharper and sharper threshold-like functions $\phi$ (**a**) and $\phi ^{-1}$ (**b**).

If the nonlinear law approaches the ideal limit characteristic, as depicted in Fig. 3a (see also the inverse characteristics in Fig. 3b), then we have the following results (derived in “Methods”).

**Figure 4:**
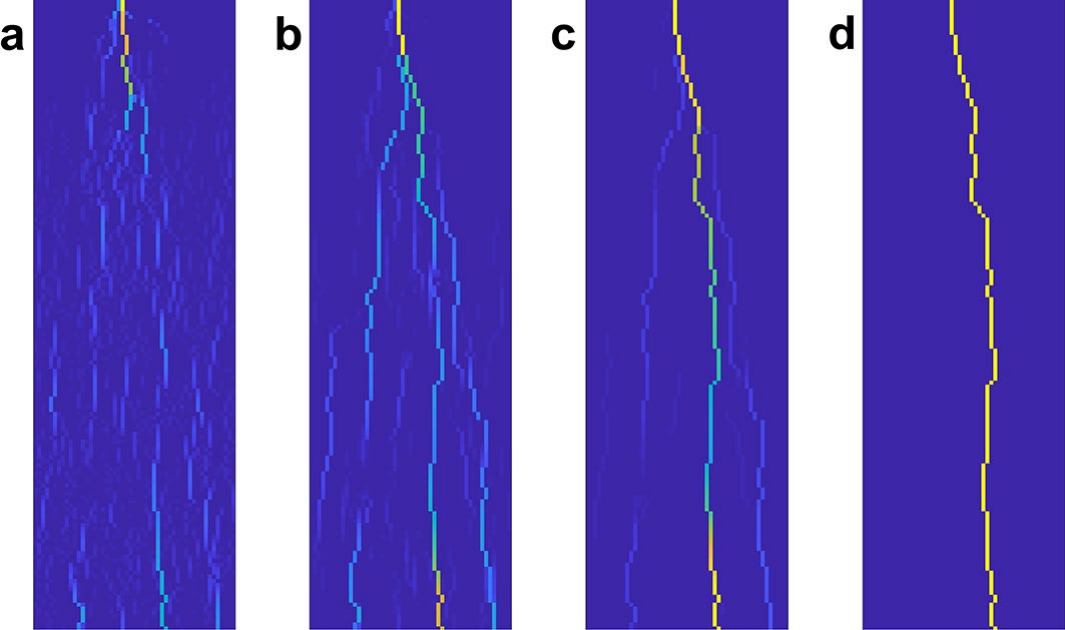
The lightning transient phases. Several branches are initially generated by the stepped leader (**a**); the stepped leader meets a connecting leader and the cloud is connected with the ground (**b**); only the main branch persists, corresponding to the optimal path w.r.t. $J^{path}_h$ (**c**); the optimal path is crossed by the long-duration current, steady state (**d**). The color map goes from blue (no current) and light blue (low intensity current) to yellow (high intensity current).

- The steady-state current distribution minimizes the cost function $J^{th}= \sum _{k} V_k |i_k|.$
- Considering the family $\mathbb {P}$ of all possible paths connecting the source node 0 to the ground node *n*, the whole injected current *d* flows along the path ${\mathcal P}^* \in \mathbb {P}$ that minimizes the associated total power, as follows:
  $$\begin{aligned} J^{path}_h=&d \sum _{k \in {\mathcal P}} V_k&\rightarrow \min \\&\text{ s.t. } \mathcal{P}\in \mathbb {P}&\end{aligned}$$
  where $k\in {\mathcal P}$ denotes that path ${\mathcal P}$ crosses link *k*.
- In the transient, the injected current starts flowing along several “tentative” branches. When one of these branches—corresponding to the minimum-threshold path—first connects to the ground (in general with the aid of a connecting leader), all the others branches are depleted, as shown in Fig. 4, obtained through simulations that faithfully reproduce this behavior.

## Discussion

The analysis of a grid circuit with capacitors and resistors having nonlinear characteristics unravels why flow phenomena such as lightning tend to concentrate the whole current flow along a single path, despite the availability of several admissible routes: this phenomenon is due to the threshold mechanism associated with the dielectric rigidity. In fact, for a nonlinear resistive network model, the solution of the flow equations minimizes a convex functional. In the special case of linear resistance, this functional is the dissipated power. In the case of threshold-like nonlinear characteristics, we have proven that the minimized functional is the sum of the currents along the links, weighted by the link threshold; hence, all the current eventually flows through the global minimum path if the links connecting the grid nodes are weighed by their local dielectric rigidity. In real situations, the dielectric rigidity can vary randomly and drastically in space, being a function of the local humidity, temperature, pressure and pollution. This explains the seemingly random path of lightning: such a randomness is due to the gas current condition, because the lightning actually searches the optimal path.

The threshold model, including capacitive effects among nodes, faithfully describes also the transient and our simulations reproduce the behavior described in^2^ and analyzed in^5,15^.

Our model does not take into account inductive effects, considered by some authors in the return stroke analysis^2^, pp. 169–170, but this does not invalidate our results because: (1) the minimum path analysis is carried out at steady state, ${\dot{v}}=0$, when the inductances are equivalent to shortcuts; (2) the return stroke starts when the stepped leader has reached the ground, hence the path has been already “decided”.

Our analysis reveals that lightning is one of the many phenomena in nature where a spontaneous optimization appears to take place^17–19^, leading to the most efficient path choice^20^. In our lightning discharge model, the resulting steady-state current flow is *globally* optimal, even though the current flow is *locally* determined on the basis of the impedance characteristic of each single link.

## Methods

The network is modeled as a grid graph with $n+1$ nodes and *m* links (more details on our model and our assumptions are in the SI Sect. 1.1). Node 0 represents the node where a current *d* is injected. The *k*th electric component is associated with link *k* connecting nodes *h* and *j*. Its impedance is given by the parallel connection of a capacitance and a possibly nonlinear resistor (cf. Fig. 1), so that the current $i_k$ flowing through the component can be written as

2 $$\begin{aligned} i_k = \phi _k \left( v_h-v_j\right) + \frac{d}{dt}\left[ C_k (v_h-v_j)\right] , \end{aligned}$$

where $v_h$ and $v_j$ are the potentials at the terminals *h* and *j*, while $\phi _k(\cdot )$ is the resistor *current–voltage characteristic function* and $C_k$ is the capacitance.

Function $\phi _k(\cdot )$ is any odd increasing locally Lipschitz, or twice differentiable, function (the case in which $\phi _k(\cdot )$ is non-decreasing only is considered in the SI Sect. 1.1). In the case of a linear resistor, $\phi _k=(v_h-v_j)/R_k$. We are interested in threshold-like characteristic functions whose value is close to zero in an interval $[-V_k,V_k]$ and becomes very large if the voltage crosses the threshold value $V_k$. In Fig. 3, the generic function $\phi$ is depicted (red curve, a) along with its inverse function $\phi ^{-1}$ (red curve, b).

Following the description of Category 1 Lightning in^2,3^, we distinguish two phases.

- First, the stepped leader “seeks the path to the ground”: the current is relatively low and the capacitance effect dominates, leading to a fast variation of the potentials at the nodes, in the transient evolution of the model.
- Then, once the stepped leader has connected the cloud to the ground, dielectric breakdown is fully developed and the long-duration discharging current is triggered. We analyze this phase assuming steady-state conditions.

The initial branching phase is by far shorter than the second phase. Indeed, only by means of special very fast video equipment the first stage can be observed, while the second one can be captured by the human eye as we commonly experience. During the second phase, the current actually varies with time but its variation rate is extremely low with respect to the first phase and then we can approximate this state as a steady state.

In both phases, we show that the presence of a threshold mechanism is crucial to enable the observed behavior: it explains both the transient evolution of the phenomenon and the achievement of a minimum-path steady-state configuration.

We analyze by simulations the initial transient (first phase), during which the path is “decided” and the currents converge to a steady-state distribution.

Let us initially show that in the second phase the system is in a steady-state, i.e., terminal potentials satisfy the condition $\dot{v}_h = 0$. In this state, the non-null currents define a single flow along the minimum path in terms of dielectric rigidity.

### Steady-state analysis: the chosen path is the minimum path

We start by showing that a threshold mechanism yields steady-state minimum-path flow.

We consider the functional

3 $$\begin{aligned} J(i) \doteq \sum _{k} f_k(i_k) \doteq \sum _{k} \int _0^{i_k}~\phi _k^{-1}(I) dI, \end{aligned}$$

where the index *k* refers to the links (see SI Sect. 1.1 for details). As a first result we have the following proposition, proven in the SI Sect. 1.3.

#### Proposition 1

Given the injected current *d*, at steady state ($\dot{v}_h=0$) the current distribution in the network minimizes the functional given by (3). If the $\phi _k$ are strictly increasing, the optimal current distribution is unique.

Note that dimensionally $J(i_k)$ is a power, since $\phi _k^{-1}(I)$ is the potential difference between two nodes connected by a link, as a function of the link current *I*, while the differential *dI* has the dimension of a current. Consistently, in the special case of linear resistances $R_k$, namely when $I=\phi _k(v) = v/R_k$ and $\phi _k^{-1}(I)=R_k I$, the function in (3) corresponds (up to the factor 1/2) to the total dissipated-power distribution in (1), which is the minimal^16^, Application 1.8, Page 15.

Here we are interested in the case in which the resistor characteristic is threshold-like. The ideal threshold function corresponding to a dielectric rigidity value $V_k$ is (see Fig. 3a, blue)

4 $$\begin{aligned} \phi ^{th}_k(v) ={\left\{ \begin{array}{ll} 0 &{}\quad \text {if }|v| \le V_k, \\ +\infty &{}\quad \text {if }v > V_k, \\ -\infty &{}\quad \text {if }v < -V_k. \end{array}\right. } \end{aligned}$$

This ideal characteristics is physically unfeasible and will not be used for our simulations. However, the corresponding optimization problem is still well defined. Indeed, the “inverse function” of $\phi ^{th}_k$ (see Fig. 3b, blue) is

5 $$\begin{aligned} g^{th}_k(i_k) ={\left\{ \begin{array}{ll} \text {any value in}~ [-V_k,V_k] &{}\quad \text {if }i_k=0, \\ +V_k &{}\quad \text {if }i_k > 0, \\ -V_k &{}\quad \text {if }i_k < 0. \end{array}\right. } \end{aligned}$$

and for this choice the functional in (3) becomes

6 $$\begin{aligned} J^{th}(i) \doteq \sum _{k} V_k |i_k|. \end{aligned}$$

The following proposition holds and is proven in the SI Sect. 1.4.

#### Proposition 2

Given the injected current *d*, the admissible (compatible with Kirchhoff’s laws) distribution of the steady-state link currents $\bar{\imath }_k^{th}$, $k=0,\ldots ,m-1$, which minimizes functional (6), corresponds to all the current flowing from the source node to the ground along a minimum-threshold path, namely a path ${\mathcal P}^* \in \mathbb {P}$ (where $\mathbb {P}$ is the family of paths from the source node to the ground) that minimizes the cost

$$\begin{aligned} J^{path}(\mathcal {P}) = d \sum _{k \in \mathcal {P}} V_k, \qquad \mathcal {P} \in \mathbb {P}, \end{aligned}$$

which is the sum of all dielectric rigidities of the links along the path.

Functional (6) is not strictly convex, hence uniqueness is not ensured (see the SI Sects. 1.1 and 1.4 for further details). However, the uniqueness assumption is *generically satisfied*; in fact, if the dielectric rigidities are randomly generated, the probability of finding two or more minimum paths with the same rigidity is zero, hence the minimum path is unique almost surely, i.e., in practice, we can assume that it is unique.

The next step is to show that the closer a characteristic function (red in Fig. 3) is to the ideal threshold $\phi ^{th}_k(v)$ (blue in Fig. 3), the closer the current distribution is to the minimum-path distribution. Given a sequence of characteristics $\phi ^{r}_k(v)$, $r=1,2, \dots$ which “converge to the ideal one” and are physically feasible, so that the corresponding steady-state solutions are uniquely defined, these steady-state solutions converge to the minimum-path distribution. This property is formalized in the following theorem.

#### Theorem 1

Consider a sequence of characteristics $\{\phi _k^r\}_{r\in \mathbb {N}}$ such that, for all *r*, the corresponding steady-state current distributions $\bar{\imath }_k^{r}$ in the links are uniquely defined. Assume that the threshold-like characteristics converge to the ideal one

$$\begin{aligned} \phi ^{r}_k(v) \rightarrow \phi ^{th}_k(v), \quad \text{ as }~r \rightarrow \infty . \end{aligned}$$

Moreover, assume that the minimum path in terms of sum of dielectric rigidities is unique. Then the link current distributions $\bar{\imath }_k^{r}$ converge to $\bar{\imath }_k^{th}$,

$$\begin{aligned} \bar{\imath }_k^{r} \rightarrow \bar{\imath }_k^{th}~\text{ as }~r \rightarrow \infty , \end{aligned}$$

namely, the current distributions converge to the one with the whole current *d* flowing along the minimum path.

Functions $\phi ^{th}_k$ are an idealized version of the gas dielectric characteristics in which the admittance is virtually zero for small voltage values and very large if the voltage is larger than the threshold value known as *dielectric rigidity*. In practice, true characteristics can be reasonably approximated^5,15^ by a continuous curve that drastically increases after the threshold. The meaning of the theorem is that, if these characteristics are sharp and close to $\phi ^{th}_k$, then the current tends to flow along the minimum path. The result does not rely on any specific characteristic model: only the property of the characteristics becoming “close to the ideal” is essential.

**Figure 5:**
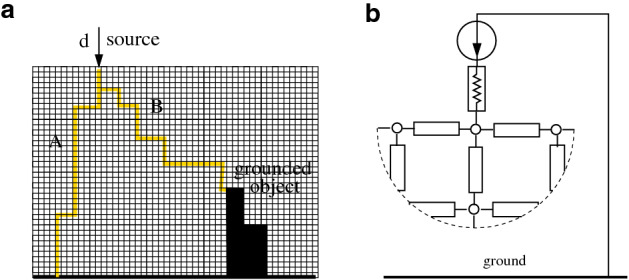
Different geometries. The model can consider, with no essential changes, different geometries. For instance, in the case of a grounded conductive object, the cells corresponding to the object are connected by very small resistance and very large capacitance values: in the left panel, lightning would choose to reach either the ground directly (path *A*) or the grounded object (path *B*) depending on which is the minimum-threshold path, essentially considering the grounded object a zero cost portion of the path (**a**). Also voltage generators instead of current generators can be considered, without any substantial change, provided that an input resistance is present (**b**).

In the model, we consider cell-to-cell capacities, but other capacities, such as capacities with respect to the ground, can be considered and the analysis remains valid, since at steady state the current through the capacities is zero. It is also fundamental to remark that the result is topology-independent: we could consider any network topology, not necessarily a grid. Also, we could consider conductive elements on the ground; in this case the lightning may find the minimum cost path as the one that connects the source to the grounded object. Some examples are in Fig. 5.

### Transient analysis: seeking the minimum path

We show here that a threshold mechanism also explains the lightning transient behavior, which can be described by the dynamic model (see the SI Sects. 1.1 and 1.2 for details)

7 $$\begin{aligned} BC B^\top \dot{v}(t) = - B\phi \left( B^\top v(t) \right) + \bar{d}, \end{aligned}$$

where *C* is the diagonal matrix including on the diagonal the capacitance at each link and $\bar{d}$ is a vector with a single nonzero entry, the first, equal to *d*, while vectors *v* and $\phi$ stack the node quantities $v_k$ and $\phi _k$. Matrix *B* is the generalized incidence matrix of the graph, formally defined in the SI Sect. 1.1. The rows of matrix *B* are associated with the graph nodes; each column corresponds to an oriented link and has a 1 entry in the position of the origin node and a $-1$ entry in the position of the destination node, while all other entries are 0. Since we assume the existence of links from the external environment, *B* is full-rank and so $BC B^\top$ is non-singular. The value of the product $BC B^\top$ is independent of the orientation chosen for the grid links, which can be consequently chosen randomly.

This system asymptotically converges to the steady-state condition $\dot{v}(t)=0$, which leads to the condition $B \phi \left( B^\top v(t)\right) = \bar{d}$ corresponding to the constraint of the optimization problem considered in the steady-state analysis. A detailed stability analysis is in the SI Sect. 1.2.

The transient analysis has been carried out via simulation, using a standard ODE solver. In particular, to numerically demonstrate the dynamic behavior of the system, we have performed many simulations for different values of the dielectric rigidity. Videos are available to display some particularly significant cases (see the Supplementary Material Movies S1–S6 for details).

**Figure 6:**
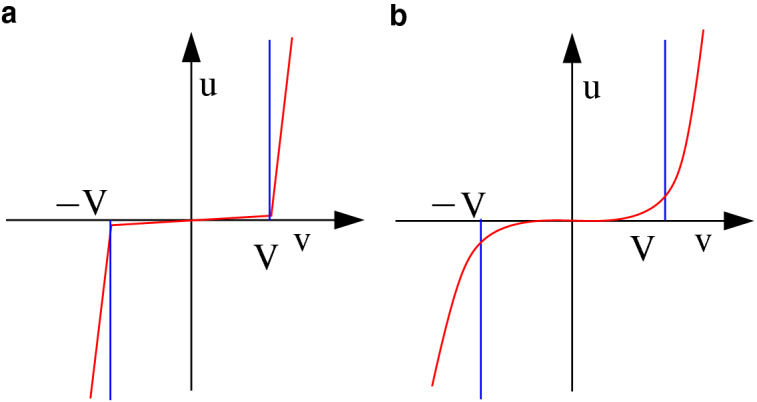
The characteristics used in simulation. The piecewise-linear in (8) (**a**) and the polynomial $(v_k/V_k)^{2r+1}$ (**b**). There is no essential difference in the final results of the simulations. For large enough values of *r*, the current follows the shortest path.

The characteristic function $\phi _k(v_k)$ can be any locally Lipschitz or continuously differentiable function that is non-decreasing and has a very high slope after the threshold. For simulations purposes, we have adopted the piecewise-linear threshold-like functions

8 $$\begin{aligned} \phi _k(v_k) ={\left\{ \begin{array}{ll} \epsilon v_k &{}\quad \text {if }|v_k| \le V_k, \\ r(v_k-V_k) +V_k \epsilon &{}\quad \text {if }v_k > V_k, \\ r(v_k+V_k) -V_k \epsilon &{}\quad \text {if }v_k < -V_k, \end{array}\right. } \end{aligned}$$

(shown in Fig. 6a), all with the same plasma conductivity $r = 800$, whereas $V_k$ has been randomly chosen for each link in the interval $V_k \in [0.5 - \delta /2, 0.5 + \delta /2]$ with uniform distribution. The variability of the dielectric rigidity is described by $\delta$, while $\epsilon$ is a very small number representing the negligible conductivity under the threshold $V_k$ (we have set $\epsilon =10^{-5}$).

Other “sharp” characteristic functions would produce the same behavior. We also simulated the system with the polynomial $\phi _k(v_k) = (v_k/V_k)^{2r+1}$ (shown in Fig. 6b), which yields comparable results for large enough *r*, as expected. Yet, the non-Lipschitz nature of the polynomial function is numerically challenging and requires large computational times and specialized integration routines for stiff systems.

In all our numerical experiments, the capacitances have been taken all equal. We have set $C_k=1$ for all *k*, without restriction, since changing the capacitance value is equivalent to scaling time, hence the steady-state value is unaffected. Fig. 4 reports four instants of the simulation with $\delta = 0.7$ (see Supplementary Material Movie S5). It can be seen that, for larger variability of the dielectric rigidity, i.e. larger $\delta$, the initial branching activity is more intense. However, the asymptotic behavior is qualitatively the same regardless of the value of $\delta$, with no exception: a single branch survives, which is numerically verified to be the minimum path in terms of total dielectric rigidity, as expected.

**Figure 7:**
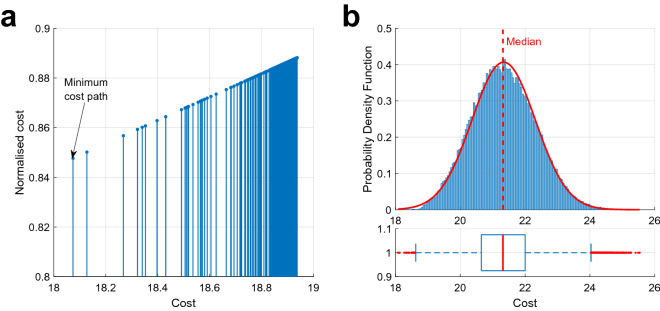
Cost analysis of randomly generated paths. Comparative analysis of the 200 paths with lowest cost (**a**) and cost statistics of all the $10^5$ randomly generated paths (**b**).

To corroborate the theory, a large number of random paths from the source to the ground have been generated on the same graph. Once again, simulation results confirm the prediction that the path chosen by the current in the simulation is always the minimum cost path. Each of the random paths is generated as follows. While traversing the nodes from the top starting point to the ground level, at each node, the current flows to the downward vertical node with probability *p*, or deviates respectively to the left node or to the right node with probability $(1-p)/2$. Fig. 7 illustrates the results obtained from the generation of $10^5$ random paths using a deviation probability $p=0.95$. Precisely, in Fig. 7a we compare the metric values of the 200 least costly paths, where the minimum-cost path corresponds to the one chosen by the current, whilst in Fig. 7b we report the distribution of the costs of all the $10^5$ randomly generated paths.

### Decentralization, topology-independence and limitations

Remarkably, the steady-state current flow in our lightning discharge model is *globally* optimal, even though the current flow is *locally* determined on the basis of the impedance characteristic of each single link. In the context of distributed flow control in networks^21^, this kind of mechanism is called *network-decentralized*^22–24^, and localized strategies have been shown to lead to a globally optimal behavior^25–31^. In the considered network-decentralized control strategy, each link locally decides how much current flows through it. This approach is completely different from Dijkstra’s decentralized minimum-path algorithm, which is based on decentralized dynamic programming techniques^32,33^ and in which the routing decision is made at the nodes: each node locally decides to which of the outgoing links an incoming unit of flow must be redirected. In our setup, a “link decision” is made: each admittance can be interpreted as an agent that locally decides how much current is allowed to flow.

Our results are independent of the topology of the network, which does not necessarily need to be a grid graph with square cells: other topologies would lead to a minimum path solution.

However, we stress that our model is far from capturing all the complex aspects of lightning. Its validity is limited to the beginning of the phenomenon, until the return stroke is triggered, because in this initial stage the path is chosen. After the stepped leader has reached the ground, the current follows the “chosen minimum path” until the end, as it is experimentally well documented (and confirmed by our simulations), because this path becomes a ionized channel with low resistance. So our model and simulations are not expected to be a faithful quantitative reproduction of the whole lightning process (including discharge endurance, power dissipation and so on), but their significance is limited to the first part. Yet, the qualitative behavior, with *the discharge following the minimum path*, has been always confirmed with no exception.

Note that we consider a constant injected current (the source turns on instantaneously at the initial time) as an assumption in our optimality theorem. If we consider an initially varying current, this can of course change the transient, but it does not change the choice of the final path, as long as the supplied current converges to a constant final value. To support this reasoning, we compared two simulations: in the first one a constant current source has been provided, whilst in the other one an increasing monotonic ramp up to the same current value has been supplied. The video obtained by these simulations (see the Supplementary Material Movie S7) reveals two different transients, which however both lead to the same final current path.

Finally, our model does not consider other aspects such as the ground currents, which are not relevant to the path choice. We have considered the so-called Category 1 Lightning, cloud-to-ground, which is the most common type of lightning; however, the model can be adapted to any type of lightning of gas discharge.

## Supplementary information

Supplementary Information.

Supplementary Video S1.

Supplementary Video S2.

Supplementary Video S3.

Supplementary Video S4.

Supplementary Video S5.

Supplementary Video S6.

Supplementary Video S7.
