# Supplementary material for: A threshold mechanism ensures minimum-path flow in lightning discharge
Source: Sci Rep. 2021 Jan 11;11:280. doi: 10.1038/s41598-020-79463-z (PMC7801417; doi:10.1038/s41598-020-79463-z)
Supplement: Supplementary file 1 — Supplementary Information. [file 41598_2020_79463_MOESM1_ESM.pdf]

A threshold mechanism ensures minimum-path flow in lightning discharge

Franco Blanchini[1], Daniele Casagrande[2], Filippo Fabiani[3],
Giulia Giordano[4*], David Palma[2], Raffaele Pesenti[5]
[1]Dipartimento di Matematica, Informatica e Fisica, Università di Udine, 33100 Udine, Italy
[2]Dipartimento Politecnico di Ingegneria e Architettura, Università di Udine, 33100 Udine, Italy
[3]Department of Engineering Science, University of Oxford, OX1 3PJ, United Kingdom
[4]Dipartimento di Ingegneria Industriale, Università di Trento, 38123 Povo (TN), Italy
[5]Dipartimento di Management, Università Ca' Foscari, 30121 Venezia, Italy
*To whom correspondence should be addressed; E-mail: giulia.giordano@unitn.it.

1 Supplementary Information

1.1 Network model and assumptions

The electrical network is associated with a graph $\mathcal{G} = (\mathcal{N}, \mathcal{L})$, where $\mathcal{L} = \{0, \dots, m-1\}$ is the set of the m links, each modeling an electric component, and $\mathcal{N} = \{0, \dots, n\}$ is the set of the $n+1$ nodes, each modeling a terminal where some components join. In particular, node n corresponds to the zero-potential ground, while at node 0 a current (or voltage) generator is applied, with its other terminal grounded, inducing an input current d that enters the network.

Consider the k-th electric component of the network, associated with the graph link $k \in \mathcal{L}$, $k = (h, j)$, which connects node h to node j: its impedance is given by the parallel connection of a capacitance and a possibly nonlinear resistor, so that the current i_k flowing through the component can be written as $i_k = \phi_k\Big(v_h - v_j\Big) + \frac{d}{dt}\Big[C_k(v_h - v_j)\Big]$ (corresponding to the admittance equation (2) in the main paper), where v_h and v_j are the potentials at the terminals h and j, while $\phi_k(\cdot)$ is the resistor *current-voltage characteristic function* and C_k is the capacitance.

We consider the *generalized node-link incidence matrix* of the graph $\mathcal{G}$, which is the matrix $B \in \{-1, 0, 1\}^{n \times m}$ obtained by assigning an arbitrary direction to each link $k = (h, j)$ of $\mathcal{G}$ and setting a 1 entry in position h (source node) and a -1 entry in position j (destination node), and zero elsewhere, in the corresponding k-th column of B, and then removing the row corresponding to node n. In particular, the m columns of B are associated with the links representing the electric components and its n rows are associated with the nodes representing terminals. Links coming from the external environment (associated for instance with an injected current) have a single nonzero entry, equal to -1, corresponding to their destination node and links going to the external environment have a single nonzero entry, equal to 1, corresponding to their source node: in our model, the connections to the external environment are the (zero-potential) ground and the source of supplied power. The following assumption holds, because we have considered the ground zero-potential node as an external node.

Assumption 1 *The network graph $\mathcal{G}$ is connected internally and connected to the external environment. As a consequence, matrix B has full row rank.*

Links are associated with the currents $i_0, \dots, i_{m-1}$ flowing through the individual electrical components, which we group in the vector $i \in \mathbb{R}^m$; nodes are associated with terminal potentials $v_0, \dots, v_{n-1}$, grouped in the vector $v \in \mathbb{R}^n$. Then,

$$i_k = \phi_k\Big(B_k^\top v\Big) + C_k B_k^\top \dot{v}\,, \tag{S.1}$$

where B_k is the k-th column of B and $B_k^\top$ its transpose, while the current balance at the node h is

$$B^h i - d_h = 0\,, \tag{S.2}$$

where B^h is the h-th row of B and d_h is the h-th element of the vector of externally supplied current.

Merging equations (S.1) and (S.2) yields the dynamics of the overall circuit $\mathcal{G}$, in terms of potentials and currents, which is described by the discretized space model [1, 2] with equations

$$0 = Bi(t) - \bar{d}, \quad i(t) = \phi\Big(B^\top v(t)\Big) + CB^\top \dot{v}(t)\,, \tag{S.3}$$

where $C = \mathrm{diag}\{C_0, C_1, \ldots, C_{m-1}\}$ is a diagonal matrix whose diagonal elements are the capacities C_k, $i = [i_0, i_1, \ldots, i_{m-1}]^\top$ is the vector whose components are the currents along the links of $\mathcal{G}$, $\bar{d} = [d, 0, \ldots, 0]^\top \in \mathbb{R}^n$ is the input current vector, $v = [v_0, v_1, \ldots, v_{n-1}]^\top$ is the vector whose components are the potentials at the nodes of $\mathcal{G}$, $\dot{v}$ is the time derivative of vector v and $\phi(\cdot) = [\phi_0(\cdot), \phi_1(\cdot), \ldots, \phi_{m-1}(\cdot)]^\top$ is the vector of the characteristic functions.

The following general assumption is considered.

Assumption 2 *Each characteristic function $\phi_k : \mathbb{R} \to \mathbb{R}$ is a possibly nonlinear odd monotonically increasing function and locally Lipschitz.*

This assumption can be weakened by requiring ϕ_k to be *monotonically non-decreasing* only.

The shape of a generic characteristic function satisfying Assumption 2 is shown in red in Fig. 3 in the main paper. The assumption implies that, for each link $k \in \mathcal{L}$, ϕ_k is invertible. Then, for each k, the function

$$f_k : y \mapsto \int_0^y g_k(s)ds\,, \tag{S.4}$$

where $g_k \doteq \phi_k^{-1}$ is the (monotonically increasing) inverse function of ϕ_k, is well defined in $(-\infty, +\infty)$.

Functions f_k are continuously differentiable. In addition, they are strictly convex, since their derivative g_k is an increasing function almost everywhere ($f_k'' = g_k' > 0$ almost everywhere; in fact, g_k' may be not defined in some isolated points, e.g. in $s = 0$ for $g_k(s) = s^{1/3}$).

If we assume that ϕ_k is non-decreasing only, then we have convexity but not strict convexity of f_k.

1.2 The dynamic model

We report here the stability analysis of the complete model (S.3), which we can rewrite in the equivalent form

$$\dot{v}(t) = -[BCB^\top]^{-1}\left[B\phi\Big(B^\top v(t)\Big) - \bar{d}\right] .$$

The stability of this type of systems has been studied in the literature [3, 4]. Consider the steady-state vector $\bar{v}$, such that

$$0 = [BCB^\top]^{-1}\left[B\phi\Big(B^\top \bar{v}\Big) - \bar{d}\right] ,$$

and denote by x the shifted variable defined as $x(t) = v(t) - \bar{v}$, whose time variation is

$$\dot{x}(t) = [BCB^\top]^{-1}B\left[\phi\Big(B^\top (x(t)+\bar{v})\Big) - \phi\Big(B^\top \bar{v}\Big)\right] .$$

Since ϕ is a vector of strictly increasing functions, we can write

$$\phi\Big(B^\top (x+\bar{v})\Big) - \phi\Big(B^\top \bar{v}\Big) = \Delta(v(x))B^\top x(t) ,$$

where $\Delta(v)$ is a diagonal matrix of strictly positive continuous functions [5] (see also [4, 6]). Hence

$$\dot{x} = -[BCB^\top]^{-1}B\Delta(v(x))B^\top x .$$

Consider the positive definite Lyapunov function candidate $V(x) = \frac{1}{2}x^\top BCB^\top x$, which is the energy stored in the capacitors. Its derivative is negative definite:

$$\dot{V}(x) = x^\top BCB^\top \dot{x} = -x^\top B\Delta(v)B^\top x < 0$$

as $x \neq 0$. This ensures asymptotic stability of the steady-state solution.

1.3 Proof of Proposition 1

We have to prove that the steady-state current distribution in the network induced by a constant current injection $\bar{d}$, achieved when $\dot{v} = 0$, namely when

$$B\phi\Big(B^\top v\Big) - \bar{d} = 0\,, \tag{S.5}$$

is indeed the current that solves the optimization problem

$$J(i_0, \ldots, i_{m-1}) \;\doteq\; \sum_{k=0}^{m-1} f_k(i_k) \to \min \tag{S.6}$$

$$\text{s.t.} \qquad Bi = \bar{d}\,, \tag{S.7}$$

where (S.7) is the flow constraint imposed by Kirchhoff's current law [7].

In view of the assumptions on ϕ_k, the function f_k defined in (S.4) is continuously differentiable with increasing derivative, hence strictly convex. Therefore, the optimization problem (S.6)-(S.7) is strictly convex and has a unique solution, achieved by applying the first order Karush-Kuhn-Tucker conditions to the Lagrangian function

$$\sum_{k=0}^{m-1} f_k(i_k) + \lambda^\top [Bi - \bar{d}]\,,$$

where $\lambda \in \mathbb{R}^n$ is the vector of Lagrangian multipliers. The derivative with respect to i must be zero, hence we get

$$\nabla f(i) + \lambda^\top B = 0\,. \tag{S.8}$$

Now, the first derivative of the elements of f is $f'_k = g_k$, which is invertible with inverse ϕ_k. As a consequence, $i_k = \phi_k\Big(B_k^\top \lambda\Big)$, hence

$$i = \phi\Big(B^\top \lambda\Big)\,. \tag{S.9}$$

The solution of the optimization problem is therefore the unique solution of the system (S.5), $B\phi\Big(B^\top \lambda\Big) - \bar{d} = 0$. Interestingly, the Lagrange multiplier vector is the steady-state potential, $\lambda = v(\infty)$.

In the case of non-decreasing functions ϕ_k, we still have convexity but not strict convexity: the result holds, but the minimizing distribution may be non-unique.

Finally note that, in the special case of linear resistances R_k, namely when $i_k = (v_h - v_j)/R_k$, the solution of the optimization problem provides (half) the minimum-dissipated-power distribution, hence

$$J^\star = \frac{P_{tot}}{2} = \frac{1}{2}\sum_{k=0}^{m-1} R_k i_k^2\,;$$

see for instance [7, Application 1.8, Page 15]. However, in the general case, each f_k can be different from the local dissipated power, which is $\frac{1}{2}P_k = \frac{1}{2}i_k\phi_k^{-1}(i_k)$. Hence, in the nonlinear case, the minimized functional is not the dissipated power as in the case of linear resistances.

1.4 Proof of Proposition 2

We have to show that the limit optimization problem

$$J^{th}(i) \doteq \sum_{k=0}^{m-1} f_k^{th}(i_k) = \sum_{k=0}^{m-1} V_k|i_k| \to \min \tag{S.10}$$

$$\text{s.t.} \quad Bi = \bar{d}\,, \tag{S.11}$$

associated with the limit characteristic ϕ_k^{th} (the limit of ϕ_k^r as $r \to \infty$), admits as its optimal solution the current distribution with all current d channeled along the shortest path.

Assume that the injected current is positive: $d > 0$ (the case $d < 0$ is identical). Let i^* denote the current distribution solving the optimization problem (S.10)–(S.11). To keep the proof simple we assume that all links in the network have been oriented in such a way that $i_k \geq 0$. This is not a restriction since link orientation is arbitrary.

Now consider the modified problem

$$\sum_{k=0}^{m-1} V_k i_k \to \min \tag{S.12}$$

$$\text{s.t.} \quad Bi = \bar{d}\,, \tag{S.13}$$

$$i \geq 0\,, \tag{S.14}$$

where the absolute value has been removed and a positivity constraint has been added.

The solution i^* of the previous problem is a feasible solution of the new problem, because its elements are nonnegative by construction. It is also optimal for the new problem. Indeed, if another solution ĩ were found with a lower cost, this would be a feasible solution also for the original problem (S.10)–(S.11) and would have a cost smaller than that of i^*.

Now note that, to solve (S.12)–(S.14), we can just take $d = 1$ and then scale the solution (by the true value $d > 0$). The proof is concluded by noticing that (S.12)–(S.14) with $d = 1$ gives the minimum cost path [7], with optimal cost $d \sum_{k=0}^{m-1} V_k$ and the whole flow through the minimum cost path. An interpretation is that the solution minimizes the overall power, measured as the product between the current flowing in a link and its dielectric rigidity.

1.5 Proof of Theorem 1

We have to prove that the steady-state solutions i^{r*} associated with the characteristic functions ϕ_k^r, which have been shown to be the minimizers of

$$J(i_0, \dots, i_{m-1}) \doteq \sum_{k=0}^{m-1} f_k^r(i_k) \to \min \tag{S.15}$$

$$\text{s.t.} \quad Bi = \bar{d}\,, \tag{S.16}$$

converge to the solution of (S.10)–(S.11) if this is unique (equivalently, the minimum path is unique).

Denote by J^r and J^{th} the cost functionals of the considered optimization problems,

$$J^r(i) = \sum_{k=0}^{m-1} f_k^r(i), \quad J^{th}(i) = \sum_{k=0}^{m-1} V_k \, |i_k| \ .$$

Since g_k^r is strictly increasing, f_k^r is strictly convex and, in turn, also J^r is strictly convex. Hence, the minimizer vector i^{r*} of (S.6)-(S.7) is unique.

Let $J^{th*} = J^{th}(i^*)$ be the optimal cost of the limit problem. For any y, $f_k^r(y) \underset{r\to\infty}{\longrightarrow} V_k\,|y|$. Then, $J^r(i) \underset{r\to\infty}{\longrightarrow} J^{th}(i)$ and, in particular, $J^r(i^*) \underset{r\to\infty}{\longrightarrow} J^{th}(i^*) = J^{th*}$. This means that the

sequence of optimal costs $\{J^r(i^{r*})\}_{r\in\mathbb{N}}$ is upper bounded by a sequence $\{J^r(i^*)\}_{r\in\mathbb{N}}$ that converges to J^{th*} as

$$J^r(i^{r*}) \leq J^r(i^*) \underset{r\to\infty}{\longrightarrow} J^{th*}\,. \tag{S.17}$$

Functionals J^r, for all r, as well as J^{th}, are radially unbounded because they are the sum of non-negative radially unbounded functions f_k^r. Then, their optimal solutions i^*, respectively i^{r*} are finite. In view of (S.17) there exists $\bar{J} > 0$ for which these solutions i^{r*} are inside the compact set

$$\mathcal{S}^{r*} = \{u \in \mathbb{R}^m \,:\, J^{r*}(i) \leq \bar{J}\}\,.$$

By construction $J^r(i)$ converges to $J^{th}(i) = \sum V_k|i_k|$, which is radially unbounded. Then, the sequence of sets $\mathcal{S}^{r*}$ is uniformly bounded in a compact set $\mathcal{S}$, hence all optimal solutions are uniformly bounded: $\{i^{r*}\}_{r\in\mathbb{N}} \in \mathcal{S}$.

We prove the convergence $i^{r*} \underset{r\to\infty}{\longrightarrow} i^*$ by contradiction. We assume that $i^{r*} \underset{r\to\infty}{\not\longrightarrow} i^*$. Negating convergence to i^* implies that there exist an open neighborhood, $\mathcal{U} \subset \mathcal{S}$, of i^* and a sub-sequence of i^{r*} that is in the complement of $\mathcal{U}$ in $\mathcal{S}$, namely in the compact set $\mathcal{S} \setminus \mathcal{U}$. In turn, this sub-sequence confined in the compact set admits a sub-sub-sequence that converges to some point $i^\circ \in \mathcal{S} \setminus \mathcal{U}$. Hence there exist a sub-sequence $\{i^{\tilde{r}*}\}_{\tilde{r}\in\mathcal{N}}$ of the original $\{i^{r*}\}_{r\in\mathbb{N}}$ that converges to some vector $i^\circ \neq i^*$, being $\mathcal{N} = \{N_1, N_2, \dots\}$ an infinite ordered set of increasing integers. All vectors $i^{\tilde{r}*}$ satisfy the constraint $Bi^{\tilde{r}*} = \bar{d}$ as they are solutions to problem (S.6)–(S.7).

Hence, also the limit vector i° does satisfy $Bi^\circ = \bar{d}$. Then, the proof can be concluded by showing that

$$J^{th}(i^\circ) \leq J^{th*}\,, \tag{S.18}$$

which is a contradiction, because it would imply that either J^{th*} is not the optimal as assumed,

or (if equality holds) that the optimization problem (S.10) has two minimum points, i^* and i°, against the uniqueness assumption.

To prove (S.18), the first step is to note that there exists a finite value g such that, for all k and for all sufficiently large r, $g_k^r(i_k) \le g$ for all i_k such that $i \in \mathcal{S}$, since $g_k^r(i_k) \underset{r\to\infty}{\longrightarrow} V_k$ and $\mathcal{S}$ is a compact set, and hence is bounded. As a consequence, for a sufficiently large r, J^r has a uniformly bounded gradient since, for all y,

$$\left|\partial J^r/\partial i_k\right|(y) = g_k^r(y) \le \max_{i_k : i \in \mathcal{S}} \{g_k^r(i_k)\} \le g\,.$$

Hence, as J^r is convex for all r, there exists a constant L such that, for sufficiently large r and for all $p, q \in \mathcal{S}$,

$$|J^r(p) - J^r(q)| \le L\|p - q\|\,.$$

Then, for $\tilde{r} \in \mathcal{N}$ sufficiently large, the following inequalities hold:

$$\begin{aligned} J^{th}(i^o) &= J^{th}(i^o) - J^{\tilde{r}}(i^o) + J^{\tilde{r}}(i^o) - J^{\tilde{r}}(i^{\tilde{r}*}) + J^{\tilde{r}}(i^{\tilde{r}*}) \\ &\le \underbrace{|J^{th}(i^o) - J^{\tilde{r}}(i^o)|}_{\to 0} + \underbrace{|J^{\tilde{r}}(i^o) - J^{\tilde{r}}(i^{\tilde{r}*})|}_{\le L\|u^o - u^{\tilde{r}*}\| \to 0} + J^{\tilde{r}}(i^{\tilde{r}*}) \\ &\le J^{\tilde{r}}(i^*) \underset{\tilde{r}\to\infty}{\longrightarrow} J^{th*}\,, \end{aligned}$$

where the last inequality and the limit come from (S.17) and the fact that $\{i^{\tilde{r}*}\}_{\tilde{r}\in\mathcal{N}}$ is a subsequence of $\{i^{r*}\}_{r\in\mathbb{N}}$ with limit i^o. Then we have shown the contradiction (S.18), which concludes the proof.

1.6 Supplementary Multimedia Files: Movies

Videos representing the transient behavior with variability δ of the dielectric rigidity equal to 0.3550 (S1), 0.4375 (S2), 0.5200 (S3), 0.6025 (S4), 0.7000 (S5), 0.7675 (S6) are available as Supplementary Material. Each video initially shows the lightning discharge, along with an artificially introduced recorded sound at the moment when the branch is crossed by the

continuing currents, and then shows a slow-motion rendering of the phenomenon. Video (S7) reports the comparison between a simulation with constant supplied current and a simulation with a current that is gradually supplied as an increasing ramp reaching the same final value. All the videos are also available on-line: `https://users.dimi.uniud.it/~franco.blanchini/Lightsim.zip`.

References

[1] Hager, W. W., Nisbet, J. S. & Kash, J. R. The evolution and discharge of electric fields within a thunderstorm. *Journal of Computational Physics*, 82(1), 193–217 (1989).

[2] Hager, W. W. A discrete model for the lightning discharge. *Journal of Computational Physics*, 144(1), 137–150 (1998).

[3] Wei, J. & van der Schaft, A. J. Load balancing of dynamical distribution networks with flow constraints and unknown in/outflows. *Systems & Control Letters*, 62(11), 1001–1008 (2013).

[4] Blanchini, F., Casagrande, D., Fabiani, F., Giordano, G. & Pesenti, R. Network-decentralised optimisation and control: an explicit saturated solution. *Automatica*, 103(5), 379–389 (2019).

[5] Blanchini, F., Franco, E., Giordano, G., Mardanlou, V. & Montessoro, P. L. Compartmental flow control: decentralization, robustness and optimality. *Automatica*, 64(2), 18–28 (2016).

[6] Blanchini, F., Casagrande, D., Fabiani, F., Giordano, G. & Pesenti, R. A network-decentralised strategy for minimum-path-flow routing. *Proceedings of the 58th IEEE Conference on Decision and Control*, 1126–1131 (2019).

[7] Ahuja, R K., Magnanti, T L. & Orlin, J.B. *Network flow* (Prentice Hall, 1993).
